# Low completion rate for the 6-months course of isoniazid preventive therapy among people living with HIV, North Eastern Uganda, 2015-2017

**DOI:** 10.11604/pamj.2024.48.122.36745

**Published:** 2024-07-19

**Authors:** Daniel Eurien, Denis Okethwangu, Dativa Maria Aliddeki, Esther Kisaakye, Joy Nguna, Lilian Bulage, Shaaban Mugerwa, Alex Riolexus Ario

**Affiliations:** 1 Uganda Public Health Fellowship Program, Ministry of Health, Kampala, Uganda,; 2 Uganda National Institute of Public Health, Kampala, Uganda,; 3 AIDS Control Programme, Ministry of Health, Kampala, Uganda,; 4 Ministry of Health, Kampala, Uganda

**Keywords:** HIV/TB, isoniazid preventive therapy, completion rates, Uganda

## Abstract

**Introduction:**

isoniazid preventive therapy (IPT) is highly effective at preventing tuberculosis among Persons Living with HIV (PLHIV). However, IPT completion rates in Uganda have not been studied. We examined completion rates for the 6-month course of IPT and factors associated with non-completion among PLHIV in northeastern Uganda.

**Methods:**

we conducted a retrospective cohort study using routinely collected program data in nine Antiretroviral Therapy (ART) sites in northeastern Uganda. The study period covered January 20 *1*5-December 20 *1*7. Non-completion was defined as failure to pick up any of the six IPT refills over 6 months. We abstracted data on IPT treatment site, IPT completion, and demographic and clinical characteristics from the IPT register and patient HIV care card. We used generalized linear regression to identify factors associated with non-completion.

**Results:**

among 543 patients who started IPT, 175 (32%) completed the full 6-month course. Among those who did not complete, 193 (52%) stopped due to drug stockouts, and 175 (48%) were lost to follow-up. Being at World Health Organization (WHO) HIV clinical stages III and IV at initiation were associated with a higher risk of IPT non-completion compared to those who were at WHO clinical staging I and II (aRR 1.4, 95%CI 1.2-1.5).

**Conclusion:**

IPT completion rate among PLHIV in northeastern Uganda was suboptimal, largely due to IPT drug stockouts. The National TB and Leprosy Program should streamline the IPT supply chain to address drug stockouts and improve completion rates.

## Introduction

In 2017, tuberculosis (TB) ranked ninth among the leading causes of death globally, and first as a leading cause of mortality from a single agent [1]. In 2017, an estimated 1.3 million deaths were attributed to TB globally among HIV-uninfected persons, and 300,000 deaths were reported among PLHIV [2]. Uganda was among the eight countries with the highest burden of HIV/TB co-infections globally [1], with a TB incidence of 201 per 100,000 among PLHIV [3,4].

To reduce the burden of TB among PLHIV, in 2008 the World Health Organization (WHO) rolled out the ‘Three I's’ strategy, comprising intensified case-finding for early treatment, isoniazid preventive therapy for persons with latent TB (to prevent progression to active TB), and TB infection control at clinical encounters [5-7]. Studies have shown that IPT reduces the risk of active TB in PLHIV by approximately one-third [8,9]. Additionally, studies in South Africa and Brazil showed a significant combined effect of antiretroviral therapy (ART) and IPT on the reduction of TB incidence, compared to ART alone [10,11]. Despite substantial evidence of the beneficial effect of IPT among PLHIV, the estimated utilization of IPT globally was only 32% among eligible PLHIV in 2013 [12].

Uganda rolled out guidelines in 2014 recommending IPT for 6 months for PLHIV with latent TB, and for children under five years of age with a history of close contact with TB [12]. However, as of 2018, the uptake of this recommendation for eligible persons in Uganda was unknown [1]. The Uganda Health Sector Annual Performance Report 2017 highlighted the lack of information on IPT completion as one of the major four challenges affecting TB planning in the country [13]. We estimated completion rates of IPT among PLHIV in northeastern Uganda who started treatment between January 2015 and December 2017 and identified factors associated with non-completion. The results may provide evidence upon which to base recommendations to public health authorities to increase the completion of IPT.

## Methods

**Study setting**: we conducted the study in the Soroti District, north-eastern Uganda, which has 8 districts [14]. Soroti municipality is the commercial center of the region hosting the Regional Hospital. The region had 41 Health Facilities providing comprehensive integrated HIV/TB services: 1 Regional Referral Hospital, 6 General Hospitals, 11 Health Center IVs, and 23 Health Center IIIs. As of 2016, northeastern Uganda had an HIV prevalence of 3.7% [15]. Soroti District began implementing the IPT program in 2015 in 20 high-volume ART clinics in collaboration with The AIDS Support Organization (TASO), a comprehensive HIV implementing partner.

**Study design:** we conducted a retrospective cohort study using routinely collected program data generated from 1^st^ January 2015 to 31^st^ December 2017 at nine ART sites in four districts in Soroti District. Completion rates of IPT among PLHIV were determined from IPT registers and HIV care clinical information was retrieved from patient ART care cards.

**Study population and sample size consideration:** we considered all records of consecutive patients in the IPT register enrolled in IPT from January 2015 to December 2017.

**Sampling procedure for health facilities:** at the time of data collection, only 20 ART sites had been accredited to provide IPT in the region during the study period, of which only nine had enrolled clients for IPT during the study period. These nine sites were included in the study and represented 1 Regional Referral Hospital, 1 General Hospital, 4 Health Center IVs, and 2 Health Center IIIs.

**Study variables, data sources, and data collection:** we abstracted data from IPT registers and patient ART care cards onto standardised forms during June 2018. Variables abstracted included facility; date of IPT initiation; demographic variables (age, sex, and marital status); IPT completion; reasons for stopping; adherence levels; and reasons for poor adherence. Variables abstracted from the ART care card included WHO clinical stage at enrolment to care; history of TB; ART adherence; and reasons for poor adherence. We defined IPT completion as a patient having picked up all six refills over the 6 months. Non-completion was defined as a patient having collected IPT pills fewer than six times over the 6 months. In Uganda, at all HIV clinic visits, the attending healthcare provider assesses adherence to ART by counting the number of pills the patient has remaining in their ART pill bottle and comparing the number of pills remaining to the number that would be remaining if the patient had taken all the pills as prescribed. These data are recorded on the patient's HIV care/ART card [16,17]. Good adherence to ART was defined as =95% of scheduled doses taken; fair adherence was defined as 80-95%; poor adherence was defined as <80%.

**Data management and analysis:** we entered the data into EpiInfo 7.2.2.6 (CDC, Atlanta, USA) and exported to Stata v. 13.1 (Stata Corp, College Station, TX, USA). We generated proportions for categorical variables and made comparisons between those who did and those who did not complete IPT using the X^2^ test for associations. Variables that produced a P value < 0.25 for X^2^ associations with IPT non-completion were included in the generalized linear model. We quantified the risk using adjusted risk ratios (aRR) with 95% confidence intervals (CIs).

## Results

**Socio-demographic and clinical characteristics of PLHIV enrolled on isoniazid preventive therapy, Soroti District, 2015-2017:** a total of 543 PLHIV were started on IPT in the nine participating health facilities during the study period. The median age was 38 years (range: 3-87). Sixty percent (326/543) of study participants were females and 76% (413/543) resided in rural areas. Adults aged 31-44 years constituted 57% (310/543); 27% (147/543) were youths aged 18-30 years. Forty-six percent (247/538) were in WHO clinical stage 1 and 13% (72/538) in clinical stages 3 or 4 at the time of enrolment to IPT. All (100%) enrolled persons were on ART at the time of IPT initiation. Seventy-seven percent (418/543) of them had good adherence to ART. Five percent (25/543) had a history of TB before initiation of IPT (Table 1).

**Table 1:** socio-demographic and clinical characteristics of people living with HIV started on Isoniazid preventive therapy, north-eastern Uganda, 2015-2017

Variable	Total (N=543)	Completion rate n (%)
**Sex**		
Male	215	73(34)
Female	328	102(31)
**Age**		
<18-35	38	12(32)
18-35	183	73(40)
≥36	322	90(28)
**Level of Health Facility**		
HCIII	75	14(19)
HCIV	165	36(22)
General Hospital	159	85(28)
Regional Referral	144	40(28)
**Residence**		
Rural	411	131(32)
Urban	132	44(33)
WHO clinical stage*		
Stage I	246	76(31)
Stage II	118	28(24)
Stage III & IV	61	7.0(12)
Adherence to ART*		
Good	459	154(34)
Fair	40	9(23)
Poor	14	6(43)
History of TB*		
Yes	75	14(19)
No	165	36(22)

* Variables contain missing values

**Isoniazid preventive therapy completion rates among PLHIV, Soroti District, 2015-2017:** of the 543 participants, only 32% (175/543) completed the full six months of IPT. Among those who did not complete, 52% (193/368) stopped due to drug stockouts, and 48% (175/368) were lost to follow-up. Among all patients who stopped treatment before completion, 32% (118/368) stopped after receiving 1 month of IPT, 42% (155/368) after 2 months, 18% (66/368) after 3 months, 5.2% (19/368) after 4 months, and 2.2% (8/368) after 5 months.

**Factors associated with non-completion of IPT among PLHIV, Soroti District, 2015-2017:** in bivariate analyses, the risk of not completing IPT among persons receiving treatment from a general hospital was 26% lower than among persons receiving IPT from a Health Center III or Health Center IV (RR 0.74, 95%CI 0.66-0.83). Patients classified as WHO clinical stage III or IV at the time of enrollment to HIV care had 40% higher risk of not completing IPT, compared with patients in WHO clinical stage I or II (RR 1.4, 95%CI 1.3-1.5). Variables such as age, sex, residence (rural or urban), adherence to ART, and history of TB were not significantly associated with adherence in the bivariate analysis (Table 2).

**Table 2:** factors associated with non-completion of isoniazid prevention therapy among people living with HIV, Soroti District, north-eastern Uganda, 2015-2017

Variable	IPT non-completion n/N (%)	cRR (95% CI)	aRR (95% CI)
**Sex**			
Male	142/215(66)	Ref	
Female	226/328 (69)	1.04 (0.92-1.17)	
**Age**			
<18	26/38 (68)	Ref	
18-35	110/183 (60)	0.87 (0.69-1.12)	
36 and above	232/322 (72)	1.1 (0.84-1.32)	
**Level of Health Facility**			
HCIII & HCIV	190/240 (79)	Ref	Ref
Hospital & RRH	178/303 (59)	0.74 (0.66- 0.83)	0.97 (0.88-1.07)
**Residence**			
Rural	280/411(68)	Ref	
Urban	88/132 (67)	1.1 (0.70-1.6)	
WHO Clinical Staging*			
Stage I &2	108/363 (68)	Ref	Ref
Stage 3&4	3/72 (4)	1.40 (1.30-1.50)	1.45 (1.20-1.50)
Adherence to ART*			
Good	305/459 (66)	Ref	
Fair	31/40 (77)	1.17 (0.81-3.7)	
Poor	8/14 (57)	0.86 (0.54-1.4)	
History of TB*			
Yes	19/25 (76)	Ref	
No	295/402 (73)	0.87 (0.34-2.2)	

* Variable contains missing values.

After adjusting for potential confounding variables, the risk of not completing IPT was not different by site. Patients classified as WHO HIV clinical stages III or IV had 4.5 times higher risk of not completing the 6-month course compared to those classified as stage I or stage II (aRR 4.5, 95%CI 1.2-1.5) (Table 2).

## Discussion

Effective prevention of progression to active TB among PLHIV is critical to addressing and ultimately controlling the TB epidemic. In our evaluation, only three of every ten PLHIV initiated on IPT completed the full 6-month course of treatment. Advanced HIV disease conferred an increased risk of failing to complete treatment, but the most common reason for stopping treatment was drug stockouts. Identification of effective interventions to improve the completion of IPT are highly needed for this population.

Drug stockouts are a challenge across the globe, particularly in low-resource settings [18-20]. Although IPT was rolled out in Uganda in 2014 in high-volume facilities, funding for medications was limited [21]. Some health facilities initiated a large number of patients on IPT anticipating stock refills that did not materialize, resulting in many patients not completing the 6 months of therapy. Our study was implemented in a routine care setting with poor patient follow-up and drug stockouts, both of which serve as barriers to completion. However, our completion rates are nearly identical to another study of IPT completion conducted between 2006 and 2008 in urban settings of central and eastern Uganda, in which patients were provided with close follow-up and there were no barriers to the supply of IPT [22]. This suggests that addressing stockouts might be necessary but not sufficient to resolve the problem of IPT completion. However, other studies in similar settings with consistently-available IPT and close patient follow-up have identified much higher completion rates, of 87-98% [23-26]. In-depth investigations to determine the root causes of poor IPT completion are paramount to improving the effectiveness of this intervention.

We noted that being in the late clinical stages of HIV was significantly associated with greater risk for non-completion. It is unclear if this was caused by their advanced disease status. Persons with advanced disease may not have been able to come to the health facility for refills as easily or if advanced HIV status is a marker for poor adherence to medications in general [27]. It is also possible that the patients died after enrollment in ART and could not complete their treatment, however, we did not have data on this [28,29]. At least one other study found no association between WHO clinical stage and IPT completion [19]. Regardless of the underlying cause, greater attention should be placed on persons at advanced WHO stages in our setting. Root cause analysis for lack of IPT completion with this particular subgroup would facilitate targeted interventions.

Of the PLHIV who did not complete 6 months of therapy, nearly half were lost to follow-up. This highlights the challenges with follow-up during rollout of IPT program. New innovative approaches like differentiated HIV care models may improve IPT completion by addressing joint barriers to IPT and HIV treatment [30,31]. In these care models, HIV-stable patients receive quarterly ART refills either in a clinic or via community adherence groups to enhance retention in care and to decongest clinics. This reduces the need for frequent travel to the clinic to pick up refills, thereby eliminating access barriers [31]. In a cross-sectional study of adult HIV patients on ART who started IPT in 5 communities in Eastern Uganda between January and April 2016, IPT completion was 72% and twice higher among those receiving IPT through Disorders of Sex Development (DSD) model compared to the traditional monthly pick-up model [32].

In 2019, the Ministry of Health launched a 100-day accelerated scale-up of IPT programming to improve implementation and increase accountability on the part of the leadership of districts engaged in mobilizing sites and addressing IPT supply chain challenges [33]. The plan seeks to enhance systems for IPT delivery, monitoring and reporting of IPT outcomes, address IPT stockouts, and improve governance and human resources among other interventions [34]. It is currently being implemented in the IPT sites countrywide and is expected to enroll 300,000 PLHIV on isoniazid preventive therapy; scale up IPT initiation by children living with HIV and with under-five TB contacts at 1,947 ART sites and ensure 100 percent completion of IPT after 100 days, both for new and previously enrolled clients [33]. The study highlights the need to capture IPT outcomes in routine electronic reporting across all health facilities. This will facilitate easy linking of patients to enable cohort monitoring and routine tracing of patients who miss scheduled visits as they are potentially at risk of not completing the full course of IPT. Finally, the supply chain of IPT must be improved to prevent drug stockouts.

**Limitations:** our study had the following limitations. We utilized routinely generated program data, which included missing data and inconsistencies in terms of documenting the reasons for non-completion. In addition, our outcome variable was based on a person picking up all six drug refills, which may not necessarily indicate that a person swallowed the drugs. This may have led to an overestimation of the proportion of people who completed the six-month course of IPT.

## Conclusion

In conclusion, the IPT completion rate in the nine ART sites in Soroti District, North Eastern Uganda was low. We recommended addressing programmatic bottlenecks, more specifically prioritizing the availability of IPT stocks. We also recommend a study to understand the reasons for poor adherence among persons with advanced HIV disease.

### What is known about this topic

- *Isoniazid preventive therapy is highly effective at preventing tuberculosis among PLHIV, reducing the risk of active TB by approximately one-third;*
- *Despite substantial evidence of IPT's benefits, the global utilization rate among eligible PLHIV was only 32% as of 2013*.

### What this study adds

- *The completion rate for the 6-month course of IPT among PLHIV in north-eastern Uganda was found to be low, with only 32% of patients completing the therapy;*
- *Drug stockouts and loss to follow-up were the main reasons for non-completion, highlighting the need for a more reliable supply chain and better patient follow-up mechanisms;*
- *Patients with advanced HIV disease (WHO clinical stages III and IV) were at a significantly higher risk of not completing IPT, indicating the need for targeted interventions for this subgroup*.
